# Post-Disaster Restoration and Reconstruction Assessment of the Jiuzhaigou Lake Landscape and a Resilience Development Pathway

**DOI:** 10.3390/ijerph20053957

**Published:** 2023-02-23

**Authors:** Liang Zhao, Gaofeng Xu, Yan Cui, Feng Kong, Huina Gao, Xia Zhou

**Affiliations:** 1 School of Urban Economics and Management, Beijing University of Civil Engineering and Architecture, Beijing 100044, China; 2 School of Architecture and Design, Beijing Jiaotong University, Beijing 100044, China; 3 National Disaster Reduction Center of China of Ministry of Emergency Management, Beijing 100124, China; 4 College of Humanities and Development Studies, China Agricultural University, Beijing 100083, China; 5 Center for Crisis Management Research, Tsinghua University, Beijing 100084, China; 6 School of Tourism Science, Beijing International Studies University, Beijing 100024, China

**Keywords:** Jiuzhaigou, restoration and reconstruction, landscape reconstruction, resilience development, “Build Back Better” (BBB), post-disaster recovery

## Abstract

The essence of post-disaster reconstruction is the restoration and rebirth of the affected areas. The earthquake hitting Jiuzhaigou was the first earthquake that had its epicenter in the World Natural Heritage located in China. Ecological restoration and landscape reconstruction are essential for the sustainable development of tourism. This study uses high-resolution remote sensing images to monitor and evaluate the post-disaster restoration and reconstruction process of the leading lakes in Jiuzhaigou. It was found that the lake water quality, vegetation, and road facilities have undergone moderate reconstruction. However, the restoration and reconstruction still faced severe challenges. The ecological environment’s stability and balance are prerequisites for the sustainable development of the World Natural Heritage sites. This paper combines the “Build Back Better” concept that advocates risk reduction, scenic spot restoration, and efficient implementation to ensure Jiuzhaigou’s restoration and sustainable development. It comes up with specific measures for the resilience development of Jiuzhaigou from the eight principles of overall planning, structural resilience, disaster prevention and mitigation, landscape facilities, social psychology, management mechanisms, policies and regulations, and monitoring and evaluation to provide a reference for the sustainable development of tourism.

## 1. Introduction

In recent years, with the frequent occurrence of natural disasters, the scenic area system has been hit hard and seriously threatened tourism development [1]. On 8 August 2017, the Jiuzhaigou earthquake was China’s first earthquake disaster, with its epicenter in the World Natural Heritage site. The magnitude was as high as seven on the Richter scale [2]. The earthquake was another major natural disaster the Jiuzhaigou scenic area encountered after the “5·12” Wenchuan earthquake in 2008 [3,4]. The superimposition of two earthquakes in ten years caused severe damage to some usual scenic spots in Jiuzhaigou’s scenic area, and the hidden dangers of geological disasters became prominent. The post-earthquake recovery and reconstruction task of Jiuzhaigou is arduous. The Sichuan Provincial Government has formulated the “8·8” Jiuzhaigou Earthquake Recovery and Reconstruction Master Plan for the post-disaster recovery and reconstruction work’s overall guidance. The planning period is 2017–2020. Unique implementation plans mainly include environmental restoration and protection, geological disaster prevention, infrastructure and public service reconstruction, urban and rural housing restoration and reconstruction, scenic restoration and upgrading, and industrial development. These planning plans guide the post-disaster restoration and reconstruction of the Jiuzhaigou scenic area [5]. Since the plan’s implementation, the scenic area has completed the ecological environment restoration in crucial areas and opened in 2020. To understand the current situation and monitor the work’s progress, it is necessary to promptly monitor and evaluate the restoration and reconstruction of the disaster area to find problems in the restoration and reconstruction and propose corresponding solutions. Restoration and reconstruction assessment of disaster areas is a comprehensive evaluation of the effectiveness in the affected areas, in order to understand the effectiveness of restoration and reconstruction implementation as well as any problems, which is conducive to better restoration and construction of the affected areas and to achieve their sustainable development [6,7].

Resilience is an emerging concept in the field of disaster management. Its application range extends from the original engineering field to ecological and social areas and has experienced engineering resilience, ecological resilience, and social resilience. These developments have gradually enriched the connotation of resilience [8,9]. Resilience in disaster management generally refers to the ability of systems and communities exposed to risks to practically resist, absorb, and adapt to threats, maintain their basic structure and functions and recover from disasters [10]. At present, resilience research in the social field focuses on the resilience of urban communities. Scenic spots are regional places with corresponding tourist facilities and tourism services in the social ecosystem. There needs to be more research on the resilience development of scenic spots [11]. Existing scenic area studies are mostly special studies for its earthquake disaster or scenic area sustainable development, this study is more systematic based on scenic area earthquake disaster and landscape analysis before and after restoration and reconstruction to propose a resilience enhancement path. The scenic spot’s resilience emphasizes the ability of the scenic spot system to resist disasters without overly relying on external forces to carry out self-regulation and recovery in combination with its own resources and environmental characteristics [12]. Scenic resilience discusses a disasters’ impact on the scenic system from an innovative perspective and provides new ideas for disaster management and sustainable development of scenic spots. Improving the resilience level of scenic spots will reduce the region’s vulnerability [13]. Establishing a framework system for the resilience development of scenic spots and exploring complementary strategies to enhance the resilience of scenic spots is conducive to promoting sustainable tourism development [14].

The Jiuzhaigou scenic area is the first nature reserve in China whose primary purpose is to protect natural scenery. It includes tourist resources such as mountain lakes, waterfalls, colorful forests, snow peaks, blue ice, and Tibetan customs. The 108 mountain lakes in the scenic area have formed the most representative lake landscape and are also the typical landscape severely damaged by earthquakes. The Jiuzhaigou earthquake destroyed the landscape in the picturesque area but also promoted the reconstruction of the landscape. For example, the earthquake caused damage to the Sparks Sea but produced a new scenic spot called Shuanglonghai Waterfall [15]. Therefore, post-disaster restoration and reconstruction of scenic spots need to focus on natural restoration, using post-earthquake geological changes to carefully cultivate new landscapes, and at the same time, through conducting scenic restoration and reconstruction monitoring and evaluation, comprehensively improving the quality of scenic restoration and reconstruction [16]. Traditionally, the monitoring and evaluation of the restoration and reconstruction of disaster-stricken areas are mainly based on tracking statistics, and there are problems such as large survey workloads and difficulties in data collection. However, remote sensing technology is more effective for monitoring and evaluating post-disaster restoration and reconstruction of particular landscape types, such as the World Natural Heritage combined monitoring. The problems found in the evaluation are conducive to studying sustainable tourism development strategies [17]. This study uses high-resolution remote sensing images to compare the state of some severely damaged lakes before and after the earthquake, analyzing the post-disaster Jiuzhaigou lake landscape reconstruction process, and combines the evaluation results to further explore the resilience development path of the scenic spot under the “Build Back Better” (BBB) concept. Finally, this study may reference the sustainable development of post-disaster tourism in the World Natural Heritage scenic area.

## 2. Materials and Methods

### 2.1. Data Sources

**Figure 1:**
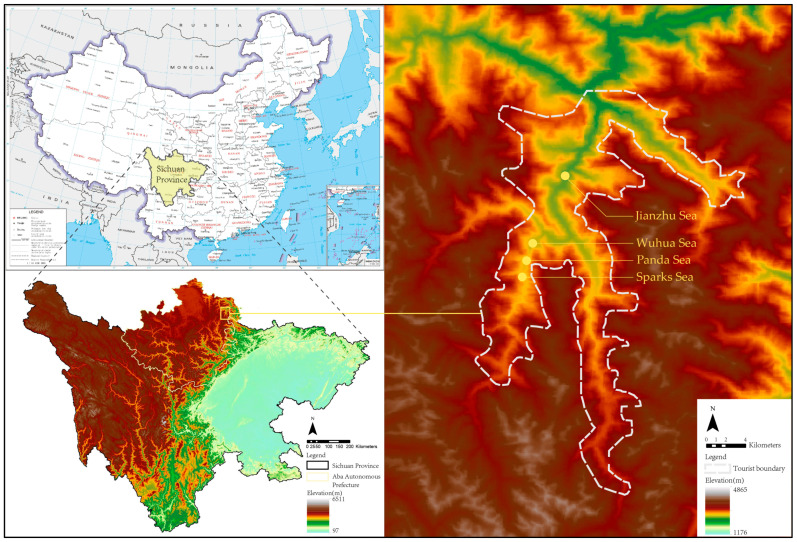
Schematic diagram of the study area.

This article takes the Jiuzhaigou scenic area, a World Natural Heritage site in the earthquake-stricken area of Jiuzhaigou on 8 August, as a case study to study its lake landscape restoration and reconstruction process and the path of resilience development. Jiuzhaigou scenic area is located in Zhangzha Town, Jiuzhaigou County, Aba Tibetan and Qiang Autonomous Prefecture, Sichuan Province, with geographic coordinates of 100°30′–104°27′ E, 30°35′–34°19′ N. It is located on the Qinghai–Tibet Plateau and the Western Sichuan Plateau, belonging to a mountainous area, a transition zone to the basin. With abundant rainfall and complex terrain, this area has frequent geological disasters, such as earthquakes, landslides, and mudslides [18,19]. In this paper, based on the damage degree and visibility of the lake landscape in the earthquake disaster, four lake landscapes, including the Sparks Sea, the Arrow Bamboo Sea, the Panda Sea, and the Wuhua Sea, were selected as the primary research objects. These lakes are primarily distributed in Shuzheng at an altitude of 2130 to 3100 m in Shu Ze Ditch and Ri Ze Ditch, as shown in Figure 1. Through spatial analysis with the help of high-resolution remote sensing images, the focus was on comparing the different states of the lake landscape before and after the earthquake and restoration and reconstruction, analyzing the impact of natural disasters and restoration and reconstruction on the lake landscape, and comprehensively exploring sustainable tourism development path.

The data used in the research mainly includes the four phases of high-resolution remote sensing images covering the Jiuzhaigou scenic area. The time phases were 1 July 2017, 16 August 2017, 19 April 2018, and 22 May 2019, and the resolution was 2 m × 2 m. See Table 1 for details of the data. The scale of the DEM data in the study area is 1:250,000.

### 2.2. Methods

#### 2.2.1. Data Pre-Processing

Through a comprehensive comparison of the four lake landscapes in the Jiuzhaigou scenic area before and after the earthquake in 2017 and the post-disaster recovery and reconstruction in 2018 and 2019, this research analyzed the damage caused by the earthquake and the post-disaster recovery and reconstruction status. This research needed to obtain various images, such as pre-disaster reference images, post-disaster loss images, and post-disaster restoration and reconstruction images, to monitor and evaluate the different stages. Remote sensing image pre-processing includes image correction and registration, band fusion, image stitching, and cropping. The regional growth method is conducted for landscape extraction and visual performance through remote sensing image interpretation and spatial analysis. GIS technology was applied on this basis, specifically for pre-disaster (1 July 2017), post-disaster (16 August 2017), and later restoration and reconstruction (19 April 2018, and 22 May 2019). The fourth phase of remote sensing images were used to study the reconstruction process of the lake landscape damaged by the earthquake to analyze the changes in various elements.

#### 2.2.2. Establishing a Landscape Classification System

Establishing a landscape classification system is the basis for image classification and pattern analysis. Due to the different regional conditions of the specific research, the landscape classification is also different. According to the study area’s particular conditions, this paper divides the land-use landscape components into forest land, waterbody, bare land, and construction land. See Table 2 for details [20,21].

#### 2.2.3. Selection and Calculation of Landscape Index

This paper’s evaluation of the Jiuzhaigou lake landscape was mainly based on the landscape’s fragmentation degree. The index selected primarily reflects the information on the fragmentation degree of the landscape, and the minimum patch area, patch density, and fragmentation index were specifically chosen for the analysis.

First, the remote sensing image were converted into the TIF format in the ENVI software in the index calculation process. After processing in Arcmap, the images were imported into the Fragstats4.2 software, and the selected index calculation performed.

(1) Patch density index

The patch density index refers to the ratio of the number of patches to the area in the study area, namely:

(1) $$PD=\sum n_{i}/A$$

where PD represents the patch density index. N represents the total number of landscape patches in the study area or the number of patches of a specific landscape patch r A represents the total study area or the area of a particular landscape patch type. The larger the PD value, the higher the degree of fragmentation.

(2) Fragmentation index

The fragmentation index refers to the degree of fragmentation of the landscape. It reflects the intensity of human disturbance on the landscape pattern to a certain extent. The calculation formula is:

(2) $$FN=\left(NP-1\right)/NC$$

where NC divides the total study area by the smallest patch area. It uses the smallest patch area as the base to reduce data changes caused by different grid sizes and because there is no village-scale topographic map. NP is the total number of various scene units in the landscape, and FN is the fragmentation degree of specific geography in the entire study area. FN ∈ (0, 1), 0 means that the landscape is not destroyed, and 1 means it is completely destroyed.

## 3. Results

### 3.1. Analysis of Lake Landscape Restoration and Reconstruction

**Figure 2:**
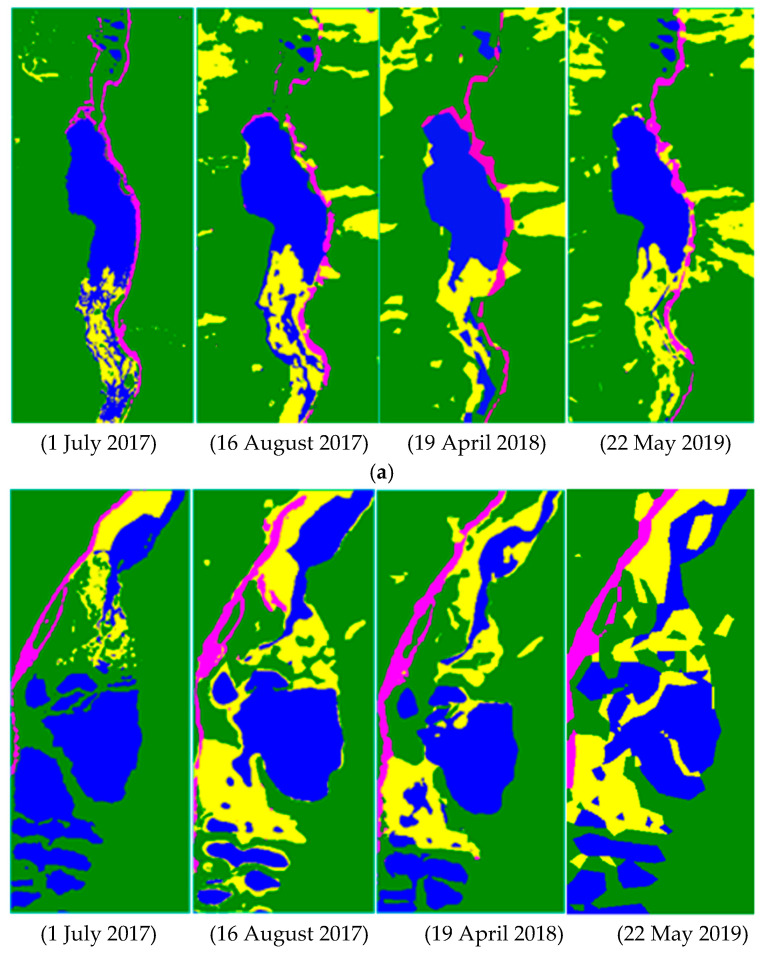

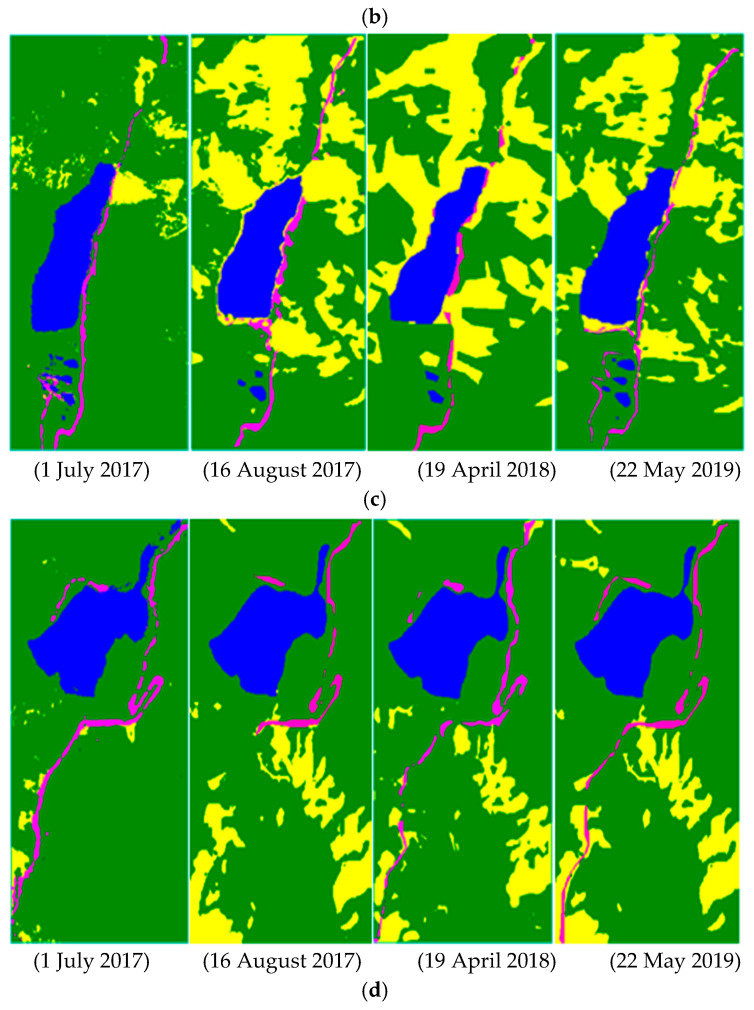
(**a**) Remote sensing images in different periods before and after the Jianzhu Sea earthquake. (**b**) Remote sensing images of different periods before and after the Sparks Sea earthquake. (**c**) Remote sensing images in different periods before and after the Panda Sea earthquake. (**d**) Remote sensing images in different periods before and after the Wuhua Sea earthquake.

Through the comparison and analysis before (1 July 2017) and after (16 August 2017) the disaster of the four lake landscapes in the Jiuzhaigou scenic area, including the Jianzhu Sea, the Sparks Sea, the Panda Sea, and the Wuhua Sea, the remote sensing images of the lake landscape was compared and analyzed. The damage was monitored and evaluated. It can be seen from Figure 2 that after the earthquake, steep slopes, and secondary disasters, such as landslides and mudslides, caused vegetation damage, and the lake water level, water quality, flow rate, and travertine deposition rate were all affected. As a result, a large amount of lake water was lost or covered by silt, increasing the silt content, turning the water body yellow, and reducing the lake landscape’s effect. Additionally, the earthquake caused damage to the vegetation in the hilly areas around the lake and the road facilities along the lake, causing heavy losses to the landscape resources. Through comparative analysis of the remote sensing images, it was found that the vegetation on the north and west sides of the Jianzhu Sea was seriously damaged, and the sightseeing roads along were also damaged to some extent. The Sparks Sea was the most severely damaged lake landscape. The south-west side of the Sparks Sea was filled with silt, causing a significant rupture, and the lake’s water level dropped considerable. The vegetation around the Panda Sea was seriously damaged, the vegetation coverage declined, the lake turned yellow, and the sightseeing road on the east side was blocked. The vegetation on the south side of the Wuhua Sea was seriously damaged, and the sightseeing road on the south side was blocked.

After further analysis of the restoration and reconstruction of the four lakes and landscapes at different times (19 April 2018 and 22 May 2019) after the disaster, it was found that the road facilities were first restored and reconstructed within eight months after the disaster, and regular traffic was restored. The Jianzhu Sea, Panda Sea, and Wuhua Sea, after the surrounding damaged roads were restored and reconstructed, still had low vegetation coverage rates. Damaged lakes, such as the Sparks Sea, had not yet been effectively repaired, and the lake landscape’s effect needed to be improved. As of May 2019, the lake landscapes of the Jiuzhaigou scenic area has been restored, and road facilities have returned to normal. The vegetation in the surrounding area has recovered more obviously. The vegetation on the north side of the Jianzhu Sea, around the Panda Sea, and the south side of the Wuhua Sea has been moderately restored.

Furthermore, the water body of the lake has been further improved. The dry lake in the south of the Jianzhu Sea has been filled. The lake, located on the southwest side of the Spark Sea, has been repaired but without its original appearance because of the severe damage. The Panda Sea and Wuhua Sea have also been moderately managed, the lake has become more apparent, and the overall landscape effect has been significantly improved.

### 3.2. Lake Landscape Composition and Cause Analysis

The Jiuzhaigou lake landscape was formed under specific geological and geographical conditions. The formation of these landscapes is affected by many factors (Table 3). The geological structure has thick limestone with good permeability, and the rock formations are compressed faults. The development of folds, fractures, and joints promotes underground water infiltration. Secondly, the height difference between the mountain peak and the valley is significant, promoting the formation of travertine deposits in the groundwater exposed in the valley under the effects of temperature and pressure. Furthermore, the CO_2_ generated after the decomposition of the vegetation around the lake promotes the increase in carbonic acid in the water. The abundant microbial photosynthesis also enables the supersaturation of calcium carbonate to form travertine deposits [22].

Additionally, lake water quality becomes better and increases the color gradation due to the high transparency of the lake water, coupled with the selective absorption and reflection of the perspective light by the grey-white travertine and yellow-green algae at the bottom of the lake. The lake water has a low sand content. There are 13 types of lake plant communities, an incomplete ecological community series, and a simple community structure (Table 4). These vegetation characteristics promote the diversification of the lake landscape [23,24].

### 3.3. Landscape Index Analysis

#### 3.3.1. Basic Landscape Parameters

It can be seen from Table 5 that during the restoration process from before the disaster to the two years after, the landscape pattern of the four lakes has undergone significant changes. After the catastrophe (16 August 2017) compared with the pre-disaster (1 July 2017), bare land in the four lake areas dramatically increased, while forest land, water bodies, and construction land decreased to varying degrees. Among them, forest land occupied the most significant area, constituting the great change between the two time points.

Further discussion on the recovery and reconstruction situation two years after the disaster (19 April 2018 and 22 May 2019) found that most areas restored regular transportation facilities within eight months. However, the vegetation coverage rate was still at a low level, due to the recurrence of mudslides, thus increasing the area of bare land. However, the vegetation coverage rate increased to varying degrees, as seen from relevant data two years after the disaster. The proportion of forest land in the Sparks Sea area increased by 8%, and more obvious restoration results have been achieved. However, there is still a certain degree of gap before the earthquake.

#### 3.3.2. Patch Density Index

Table 6 shows that the patch density indexes of different land types in the four lake landscapes after the earthquake mostly declined. One year after the post-disaster restoration and reconstruction (2018), the patch density indexes of woodland, water area, and bare land continued to decline while construction land increased. Two years after the post-disaster restoration and reconstruction (2019), the patch density indexes of various land types gradually increased. Among them, the Wuhua Sea showed more apparent changes in its landscape than the other three lakes.

#### 3.3.3. Fragmentation Indexes

Table 7 shows that in the same area and the same year, most of the four landscapes had the highest fragmentation index, indicating that the fragmentation degree was relatively strong. The fragmentation indexes of the different regions and landscape types were equal two years after the disaster (2019). There were different degrees of reduction, which means that the fragmentation decreased over time and the waters and vegetation were restored. The impact of earthquake damage and reconstruction can be seen by classifying the woodland, water area, bare land, and construction land, especially for the woodland and bare land. The water landscape of the Wuhua Sea was even restored its pre-disaster level to a level close to zero.

## 4. Research on the Resilience Development Pathway in Post-Disaster Scenic Spots under the “Build Back Better” Concept

Combined with the analysis of the landscape indexes of the Jiuzhaigou lakes before and after the disaster and before and after the restoration and reconstruction, it was found that the vegetation and water body landscape still need to be improved in the later restoration and reconstruction, so it is necessary to further explore the resilience development path of disaster-prone scenic areas. The nature of destination disasters is a crisis event. Earthquake disasters can negatively affect tourists’ emotions and attitudes, and the key to scenic resilience development is to enhance tourists’ scientific knowledge of disaster risks, thus promoting the sustainable development of scenic areas. Tourist attraction is a critical component of the tourism industry, and the resilience of scenic spots directly restricts the sustainable development of tourism [12]. After the Jiuzhaigou earthquake, the original landscape structure of the scenic area changed. The restoration and reconstruction after the disaster required using the principle of harmonious coexistence between humans and nature, emphasized by landscape ecology and reconstructing a good landscape ecosystem based on conserving the local ecological environment. In the construction process, it is necessary to integrate the natural background and retain the evolutionary characteristics of the ecological environment [25].

### 4.1. “Build Back Better” Concept and Evolution of Resilience Theory

**Figure 3:**
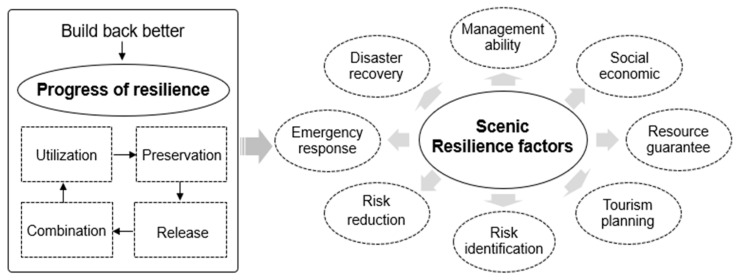
The evolution of resilience theory under the concept of “Build Back Better”.

The “Build Back Better” (BBB) concept was first widely used in the post-disaster recovery and reconstruction of the Indian Ocean tsunami in 2004 and has become an essential guiding principle for post-disaster recovery and reconstruction (Figure 3). The concept aims to create a safe and resilient community after a disaster [26]. Its international definition refers to reducing the vulnerability of disaster-stricken areas during the post-disaster recovery and reconstruction stage, incorporating concepts such as disaster risk reduction into regional development so that countries and communities can resist disasters and simultaneously improve production, life, and the ecological environment [27]. Post-disaster recovery and reconstruction are not simply to restore the disaster area to its pre-disaster state but to repair the damage and, at the same time, make up for the existing issues and reduce the vulnerability of the disaster area so that losses can be reduced in the event of another disaster [28]. The concept of “Rebuild Better” emphasizes creating sustainable communities through a series of actions after the disaster, including improving the social, economic, and environmental factors and enhancing the resilience of the disaster area [29,30]. The concept of BBB was widely practiced in the reconstruction of Sri Lanka and Indonesia’s Yaqi shelters after the Indian Ocean tsunami, suggesting that “safer” should be given priority among the many goals contained in “Better” [31]. Implementing the “Rebuild Better” strategy depends on the specific improvement goals. Based on this principle, guiding the recovery and reconstruction of the scenic spot will help solve the current dilemma.

**Figure 4:**
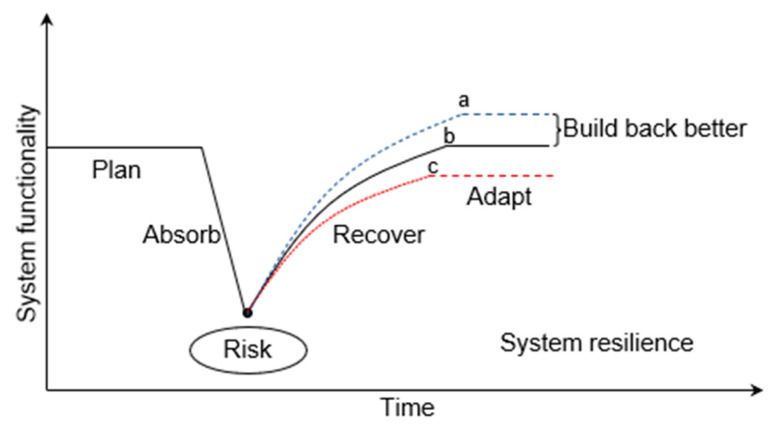
Resilience management framework centered on disaster risk analysis. (a. The post-disaster state is better than the pre-disaster state; b. The post-disaster state is equal to the pre-disaster state; c. The post-disaster state is weaker than the pre-disaster state).

The concept of better reconstruction has promoted the development of resilience theory, and traditional resilience has transformed “balanced” to “adaptable” [32]. Resilience derives from original mechanical engineering to ecology, social science, and other fields and is now widely used in disaster risk management [33]. Engineering resilience emphasizes the restoration of the system to its initial steady state after being disturbed; ecological resilience underlines the transformation of a new stable condition after being disturbed; evolutionary resilience abandons the pursuit of a constant shape and believes that resilience is an attribute of the system itself, which will undergo internal dynamic evolution regardless of external disturbances. Evolutionary resilience is based on the “adaptive cycle theory”. It proposes that the development of system resilience will go through the stages of utilization, preservation, release, and reorganization, emphasizing that the system is disturbed through continuous adaptation and learning to obtain reconstruction opportunities in the reorganization stage and enter the utilization stage again to achieve the adaptive cycle, as shown in Figure 4 [34]. Evolutionary resilience believes that resilience is a dynamic system attribute related to the ability to continuously adjust, focusing on responding to disaster risks during the system’s evolution to realize the co-evolution of the system and the external environment and “bounce to a better state” [35]. Evolutionary resilience is in line with the concept of “Rebuilding Better”, emphasizing the exploration of the fundamental laws of social ecosystems from the perspective of evolution. The principles of risk reduction, efficient execution, and scenic restoration based on the concept of “Rebuild Better” will promote the better development of scenic areas based on evolutionary resilience.

### 4.2. Research on the Path of Resilience Development in Scenic Spots after the Disaster

Scenic spots are an indispensable part of the social ecosystem. Scenic resilience is an inherent ability of a system. It focuses on describing the system’s self-organization, learning, and adaptability, as the stable state of the system is dynamically changing [36]. The exploration of the path of resilience development of scenic spots after disasters is the foundation for the successful restoration and reconstruction of scenic spots and the external guarantee for promoting the sustainable development of tourism. Since the Jiuzhaigou scenic spot contains multiple Tibetan villages and the research on community resilience is relatively mature, the resilience development of the scenic spot needs to be focused on the construction of community resilience based on the concept of BBB and the resources and environment characteristics to explore the resilience development path of the scenic spot [37]. In addition, as a World Natural Heritage site, tourists want to see the original appearance of the Jiuzhaigou scenic area. If the scenic area is rebuilt blindly after the disaster, the ecosystem’s destruction and the natural environment’s deterioration will only increase. The landscape changes caused by the earthquake will reduce the ecology of the scenic area. The adaptive capacity of the system and the homogeneity of human-made landscapes will further increase the vulnerability of the scenic site. Therefore, the restoration and reconstruction process of the scenic area should focus on natural restoration, supplemented by manual intervention, and actively explore the path of resilience development of the scenic area [38,39]. The resilience construction of scenic spots under the concept of BBB requires full consideration of the frequency and intensity of various random disturbances to ensure the adaptability of scenic spots and to predict and prepare for future shocks to innovatively design scenic spots.

**Figure 5:**
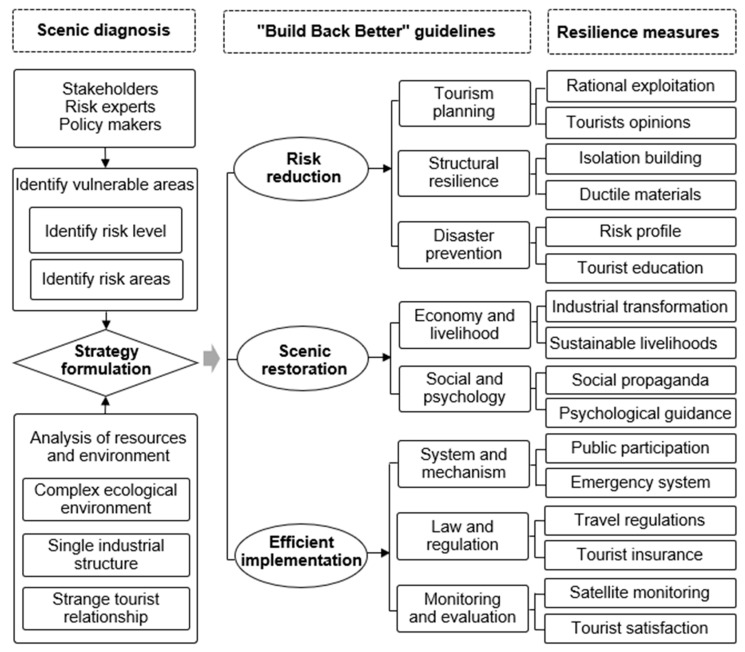
The basic framework of resilience development of scenic spots post-disaster under the concept of “Build Back Better”.

Most World Natural Heritage scenic spots are strong tourist attractions due to their rich species and unique environment. Still, they face problems such as ecological fragility and frequent disasters [40]. Compared with other scenic spots, World Natural Heritage sites have the advantage of resilience construction in post-disaster recovery and reconstruction. First, the natural attributes of the World Natural Heritage tourism resources are more robust, and many supporting facilities make them less artificial. Secondly, the evolution of the internal ecosystem in scenic areas caused by natural disasters will also promote new tourism resources. These advantages provide accessibility for exploring the post-disaster resilience development mechanisms of the Jiuzhaigou scenic area. The concept of “Reconstructed Better” and evolutionary resilience theory require attention for the short-term performance of the ecosystem post-disaster and the medium- and long-term evolution of the system [41]. Research on the resilience development path of scenic spots after disasters first requires problem diagnosis and the identification of vulnerable areas through surveys and interviews with stakeholders, disaster risk experts, and policymakers to understand the disaster risk level and the affected areas. Secondly, it integrates the complex ecological environment, frequent disaster risks, and other resource and environmental characteristics to provide a reference for exploring the resilience development mechanisms of the scenic spot. Combining the problems found in the previous diagnosis and based on the concept of “Reconstructing Better”, specific measures for the resilience development of scenic spots can be proposed from risk reduction, efficient execution, and scenic restoration (Figure 5).

#### 4.2.1. Risk Reduction

Risk reduction is an essential prerequisite for the post-disaster recovery and reconstruction of the scenic area, especially for disaster-prone scenic areas. The post-disaster reconstruction process should focus on the development of risk prevention measures in conjunction with the problems identified in the monitoring and assessment, including the planning preparation, structural resilience, and emergency response capabilities included in this level. The planning preparation is the guidance for the entire post-disaster recovery and reconstruction process. Improving structural resilience is a hardware measure to reduce risks. Emergency response capability is software support to reduce the risk [42]. First, a risk identification must be conducted through professional risk assessment agencies, and risk-prone areas must be avoided throughout the overall planning of post-disaster recovery and reconstruction to reduce the probability of disasters. In particular, it is necessary to establish emergency shelters based on the complex characteristics of the ecological environment of the scenic area and to make improvements to site selection and land utilization of the disaster area [43]. Second, the construction infrastructure of scenic spots needs to be scientifically designed to improve the earthquake resistance of buildings. Improving the structural resilience of project facilities in post-disaster recovery and reconstruction is an important measure to avoid losses in the next disaster [44]. Third, the emergency response capabilities of scenic spots include disaster monitoring and early warning capabilities, disaster defense capabilities, response and rescue capabilities, and management support capabilities. Specifically, the emergency response capabilities of scenic spots must be improved by increasing early warning facilities and carrying out emergency education [45].

#### 4.2.2. Scenic Restoration

A scenic area is an independent unit of post-disaster recovery and reconstruction. From the perspective of the social ecosystem, the development of resilience construction can ensure the sustainability of the scenic area’s recovery. Under the concept of BBB, resilience building is the most practical way to recover communities after a disaster. It can improve the ability of communities to return to normal conditions and adapt to the new environment after a disaster. Resilience building needs to focus on the unity of the external and internal. First, the society and residents’ psychology in the disaster-stricken area are the interior elements of resilience building. By explaining the disaster risk in the scenic area, the panic caused by the disaster can be reduced. In addition, emergency education is used to improve disaster prevention capabilities, and psychological assistance is provided to alleviate the psychological anxiety of the staff in the scenic area [46]. Second, the restoration of the scenic environment mainly refers to the restoration of the tourist landscape facilities, including landscape restoration and supporting facilities. Landscape restoration primarily focuses on natural restoration while paying attention to the cultivation of black tourism. Supporting facilities mainly include infrastructures such as road traffic and service facilities such as catering and accommodation. Thirdly, economic livelihood restoration will improve the resilience level of the financial system in the disaster area by adjusting the industrial structure under the resources and environment characteristics in the disaster area. It is also necessary to improve the poverty alleviation function of the restoration and reconstruction project, which is conducive to lessening “disaster-induced poverty” and “returning to poverty” [47].

#### 4.2.3. Efficient Implementation

The efficient implementation of the restoration and reconstruction of the scenic spot needs a scientific organization and management mechanism and a sound policy and regulation system. First, the government plays a leading role in the restoration and reconstruction of Chinese scenic spots, and in the organization, implementation, supervision, and evaluation. The development of the organization and management of the restoration and reconstruction of scenic spots has promoted the transition from a single subject to multiple subjects in the cooperative mechanism of participating issues [14,30]. The restoration and reconstruction of disaster-stricken areas should follow people-oriented principles and explore the establishment of planning consultation and deliberation, democratic decision-making, and fair participation, promoting the transition from “blood transfusion” to “hematopoiesis” in the disaster area. Second, in the restoration and reconstruction of scenic spots, supporting policies and regulations need to be formulated to restrict the responsibilities of different subjects and guarantee their participation rights [48]. Tourism culture and emergency management departments need to develop policies and regulations on finance, taxation, land, industry, and government agencies at all levels to refine various policies following actual local conditions to promote the efficient and stable implementation of the restoration and reconstruction of scenic spots [49].

## 5. Discussion and Conclusions

### 5.1. Discussion

#### 5.1.1. The Jiuzhaigou Scenic Area after the Disaster Restoration and Reconstruction of the Dynamic Monitoring Approach Based on the More Diversified Actual Needs

The Jiuzhaigou scenic area is high in the south and low in the north, with deep valleys and vast differences in elevation. There are problems, such as an extensive survey workload and difficulties in data collection for post-disaster recovery and reconstruction monitoring. The high-resolution remote sensing satellite images in this paper facilitate the monitoring of post-disaster recovery and reconstruction to a certain extent but monitoring the lake landscape and other elements need to be further combined with manual field surveys and relevant instruments for water quality testing. At the same time, it is necessary to conduct more microscopic monitoring and identification with the help of drones for areas that are difficult to cover by high-definition remote sensing satellite images. It is necessary to combine various forms of field exploration and sensor monitoring to identify landslides and other potential mountain geological disaster sites and the composition of biodiversity after the disaster. In this paper, post-disaster restoration and reconstruction monitoring methods were proposed. Based on these methods, research can combine with actual cases to verify the various methods’ effectiveness and suitable environments and finally provide technical guidance for the dynamic monitoring of post-disaster restoration and reconstruction in disaster-prone scenic areas.

#### 5.1.2. The Jiuzhaigou Scenic Area Post-Disaster Recovery and Reconstruction Effect Evaluation System Refined Based on Land Type

The Jiuzhaigou scenic environment is characterized by a complex ecological environment and frequent disaster risks, and the earthquake caused significant damage to the surrounding vegetation, water bodies, and tourism facilities. Through restoration and reconstruction, the fragmentation indexes of the different landscape types have been reduced by differing degrees, and the landscape has been moderately restored. However, the vegetation cover in some areas needs to be improved. By analyzing remote sensing images of the Jiuzhaigou scenic area, it is possible to understand the shortcomings of the restoration and reconstruction of forest land, water bodies, bare land, and construction land. Nevertheless, due to the differences between the different land types, a more specific evaluation system can be constructed by combining various types of land in order to propose targeted improvement measures. In addition, there is a need to balance the relationship between ecological restoration and the repair of tourism facilities post-disaster [50]. The construction of external tourism facilities has, to a certain extent, contributed to the challenges of ecological restoration in the scenic area post-disaster, and subsequent evaluation needs to consider the potential impact of these facilities on the ecological environment. This study focused on the restoration and reconstruction analysis in conjunction with the changes in the lake landscape. Specific evaluation of the restoration and reconstruction effects of the different land types, such as natural vegetation and tourism facilities, is pending to provide a more systematic understanding of the effectiveness of post-disaster restoration and reconstruction.

#### 5.1.3. Geological Disaster-Prone Scenic Areas Need to Integrate the Synergistic Relationship between Post-Disaster Recovery and Reconstruction and Development Revitalization

The Jiuzhaigou scenic area experienced the “5·12” Wenchuan earthquake in 2008 and the “8·8” Jiuzhaigou earthquake in 2017, and the two consecutive earthquakes have made the geological hazards of the scenic area more prominent. In this process, it is necessary to coordinate the intrinsic links between restoration and reconstruction and development revitalization, ensuring that the limited funds and materials are sufficient to complete restoration and reconstruction while promoting the development and revitalization of scenic spots to the greatest extent. This study combined the post-disaster reconstruction experience of some scenic spots in the “5·12” Wenchuan earthquake. It attempted to build a World Natural Heritage scenic spot with more tourism and ecological value after the disaster by integrating the recovery and reconstruction into developing and revitalizing scenic spots. If the scenic area is blindly rebuilt after the disaster, the deterioration of the scenic ecosystem will only intensify. The scenic area’s process of restoration and reconstruction should be based on natural recovery, supplemented with artificial intervention, and to explore the scenic area’s mode of restoration and reconstruction. There needs to be more research on the relationship between restoration and reconstruction and the development and revitalization of scenic spots. The post-disaster restoration and reconstruction planning of World Natural Heritage scenic spots can be in conjunction with the concept of “black tourism”. This strategy focuses on the ecological planning of black tourism resources to meet the emotional experience of tourists to memorable scenes such as earthquake disasters and environmental evolution. Integrating recovery and reconstruction with development and revitalization through various forms can realize the efficient use of resources and the sustainable development of the region.

#### 5.1.4. Post-Disaster Resilience Development Mechanisms of Geological Disaster-Prone Scenic Areas Needs to Be Further Transformed with Regional Characteristics

The dynamic monitoring and comprehensive evaluation of the post-disaster restoration and reconstruction of the Jiuzhaigou scenic area are conducive to exploring the resilience development mechanisms of geological disaster-prone scenic areas. Due to differences in World Natural Heritage scenic areas in various types of geological hazard-prone areas, it is necessary to make appropriate adjustments to the resilience development measures proposed in this study. Furthermore, to meet the development needs of specific scenic areas, the resource environment and disaster risk characteristics of the scenic areas need to be taken into account. Jiuzhaigou’s remote sensing image analysis showed that the lake water body, surrounding vegetation, and supporting facilities still need to be improved. The development mechanisms need to be optimized in post-disaster restoration and reconstruction. This paper’s “BBB” principle provides the basic idea for post-disaster scenic resilience development, of which risk reduction is the most fundamental principle. However, different types of scenic areas need to further explore the specific measures and sequence of resilience development in combination with their regional characteristics. In the future, the development of resilience development measures for scenic spots in geological disaster-prone areas needs to follow the concept of multi-body participation and propose more innovative guiding measures for resilience development with the perspective of resilience theory combined with the regional resources and environment characteristics [51].

### 5.2. Conclusions

The restoration and reconstruction of the disaster area is a long-term project. Although the essential restoration and reconstruction tasks were completed after the Jiuzhaigou earthquake, there are still many restoration and reconstruction processes to complete. The study took the lake landscapes as the research object, analyzed the impact of earthquake disasters and the effect of restoration and reconstruction and put forward a resilience development mechanism of post-disaster scenic spots based on the BBB principle based on the evolutionary resilience perspective. The study used high-resolution remote sensing images to analyze the changing process of the Jianzhu Sea, Sparks Sea, Panda Sea, and Wuhua Sea lake landscapes. By comparing remote sensing images at different periods before and after the disaster, it found that the earthquake impacts the lake landscape. After restoration and reconstruction, the water quality of the damaged lake, surrounding vegetation, and road facilities have been improved in response. However, the restoration status of vegetation and construction land around different lake landscapes varies, and only moderate landscape reconstruction has been carried out. The recovery and reconstruction monitoring can be carried out more macroscopically with the help of high-resolution remote sensing images, pending further combination with field exploration to understand the specific recovery situation. In general, the overall landscape effect still needs to be fully restored. In fact, the monitoring and assessment of lake water quality and vegetation diversity in this study can be further assisted by integrating air-space and space monitoring technologies in the future. This will more realistically reflect the restoration and reconstruction situation, and at the same time allow more targeted exploration according to the resource and environmental characteristics of specific affected areas to propose effective measures suitable for the resilience development of different types of scenic areas. To ensure better post-disaster recovery and reconstruction, and to achieve long-term development in the later period of the scenic spot, the research was based on the three principles of risk reduction, scenic restoration, and efficient implementation advocated by BBB. Combined with the evolutionary resilience theory, the overall planning, structural resilience, disaster prevention and reduction, landscape facilities, social psychology, management mechanisms, policies and regulations, monitoring, and evaluation, could be organized to propose specific measures for the resilience development of scenic spots after the disaster to guide the sustainable development of tourism. Post-disaster restoration and reconstruction is a long-term systematic project.

After the Jiuzhaigou Scenic area completed the three-year essential restoration and reconstruction task, the hidden dangers of geological disasters still exist. Combined with the problems found in the monitoring and evaluation of scenic restoration and reconstruction, the proposed scenic resilience development path plays an important role in promoting the sustainable development of scenic areas. The restoration and reconstruction will also have a particular impact on the ecological environment. The rapid increase in tourists in recent years has led to increased algae and sediments in the lake, the degradation of travertine, and increased threats to biodiversity. It is necessary to research the development of tourism resilience based on the concept of BBB to promote tourism in scenic spots to return to their original levels and ensure sustainable tourism development in scenic spots. As a World Natural Heritage site, the destruction of the ecological environment of the Jiuzhaigou scenic area is equivalent to the destruction of the economic sources of production in the region. After a disaster, the restoration and reconstruction of the scenic area must also consider the comprehensive unity of the social, economic, and ecological benefits. Traditional resilience research focuses on urban planning, engineering, and ecology. The resilience research of post-disaster recovery and reconstruction from disaster risk management has only come into view in recent years. It is imperative to explore the development path of tourism resilience based on BBB by analyzing the problems and experiences in restoring and reconstructing the Jiuzhaigou scenic area.

## Figures and Tables

**Table 1:** Data selection for remote sensing monitoring and evaluation of the lake landscape in the Jiuzhaigou scenic area.

Data Time	Remote Sensing Satellite	Sensor	Track Number	Sensor Resolution	Width
1 July 2017	GF2	PMS2	L1A0002453010	Full color 0.8 m, multi-spectral 3.2 m	45 km
16 August 2017	GF1	PMS2	L1A0002544376	Full color 2.0 m, multi-spectral 8.0 m	60 km
19 April 2018	GF1	PMS2	L1A0003131349	Full color 2.0 m, multi-spectral 8.0 m	60 km
22 May 2019	GF1B	PMS	L1A1227637059	Full color 2.0 m, multi-spectral 8.0 m	60 km

**Table 2:** Landscape classification system and description.

Type Code	Landscape Type	Description
1	Woodland	Natural and artificial forest land where trees, shrubs, and shrubs grow, with good vegetation coverage
2	Waterbody	Naturally formed or artificially excavated land for water bodies, including rivers and lakes.
3	Bare land	No vegetation, including bare soil and rock on hillsides and riverbanks
4	Construction land	Artificial buildings, mainly including transportation land and open spaces

**Table 3:** Scenery analysis of damaged lakes in Jiuzhaigou.

Name	Tectonic Trace	Karstification	Gravitational Process	Water Recharging	Lake Water Excretion
Jianzhu Sea	Rize Hotel anticline, Jiuzhaigou fault, Yingzhaodong east–west strike fault, Jiuzhaigou fault	Travertine accumulation, biokarst	Collapse, mudslide	Rainfall, upstream surface runoff, groundwater	Evaporation, discharge downstream through surface runoff, seepage loss from underground
Sparks Sea	Jiuzhaigou fault, Zechawa fault	Travertine accumulation, biokarst	Mudslide	Rainfall, upstream surface runoff, groundwater	Evaporation, discharge downstream through surface runoff, seepage loss from underground
Panda Sea	Panda Sea Anticline, Jiuzhaigou Fault	Travertine accumulation	collapse	Rainfall, upstream surface runoff, groundwater	Evaporation, discharge downstream through surface runoff, seepage loss from underground
Wuhua Sea	Wuhua Sea syncline, Jiuzhaigou fault, Wuhua Sea fault	Travertine accumulation	Landslides, mudslides	Rainfall, upstream surface runoff, groundwater	Evaporation, discharge downstream through surface runoff, seepage loss from underground

**Table 4:** Lake plant communities and their distribution in Jiuzhaigou.

Community Type	1	2	3	4	5	6	7	8	9	10	11	12	13
Jianzhu Sea	√		√		√		√		√	√			
Sparks Sea							√						√
Panda Sea			√				√						
Wuhua Sea	√			√		√	√		√	√	√		

**Table 5:** Comparison of the area changes in different periods before and after the earthquakes (km^2^).

Land Types	Lake Scenery	1 July 2017	16 August 2017	19 April 2018	22 May 2019
Woodland	Jianzhu Sea	112.74	95.61	100.88	102.99
	Sparks Sea	32.39	26.01	26.55	31.03
	Panda Sea	85.76	59.99	61.29	66.69
	Wuhua Sea	71.27	65.92	65.32	65.48
Water area	Jianzhu Sea	16.59	18.37	17.76	15.09
	Sparks Sea	12.05	11.24	11.94	10.84
	Panda Sea	10.06	9.69	8.68	10.41
	Wuhua Sea	9.08	8.70	8.90	8.70
Bare land	Jianzhu Sea	3.99	19.32	15.68	18.97
	Sparks Sea	2.75	9.46	9.00	6.00
	Panda Sea	5.20	32.70	32.37	25.53
	Wuhua Sea	1.39	11.29	8.75	11.00
Construction land	Jianzhu Sea	3.46	2.97	4.36	3.66
	Sparks Sea	1.21	2.02	2.12	2.23
	Panda Sea	2.16	0.61	1.64	2.16
	Wuhua Sea	2.74	1.43	2.20	2.16

**Table 6:** Comparison table of the patch density indexes in different periods before and after the earthquake (km^2^).

Land Types	Lake Scenery	1 July 2017	16 August 2017	19 April 2018	22 May 2019
Woodland	Jianzhu Sea	29.97	27.15	10.10	24.16
	Sparks Sea	41.31	16.41	20.16	27.94
	Panda Sea	31.98	21.36	15.39	20.99
	Wuhua Sea	30.77	11.45	11.74	12.60
Water area	Jianzhu Sea	49.71	16.88	8.65	11.37
	Sparks Sea	59.90	34.88	24.19	21.96
	Panda Sea	22.29	4.85	2.89	6.68
	Wuhua Sea	13.02	1.15	1.17	1.15
Bare land	Jianzhu Sea	57.02	47.70	18.75	45.48
	Sparks Sea	59.90	162.08	40.31	35.93
	Panda Sea	70.75	47.57	16.35	57.25
	Wuhua Sea	60.36	30.92	51.66	25.19
Construction land	Jianzhu Sea	19.74	29.35	8.65	9.95
	Sparks Sea	12.39	34.88	4.03	5.99
	Panda Sea	9.69	0.97	15.39	10.50
	Wuhua Sea	52.08	10.31	24.66	16.03

**Table 7:** Comparison of fragmentation indexes in different periods before and after earthquakes (km^2^).

Land Types	Lake Scenery	1 July 2017	16 August 2017	19 April 2018	22 May 2019
Woodland	Jianzhu Sea	0.0001	0.0002	0.00062	0.0002
	Sparks Sea	0.0001	0.0001	0.00163	0.0005
	Panda Sea	0.0000	0.0002	0.00130	0.0003
	Wuhua Sea	0.00004	0.00007	0.00007	0.00008
Water area	Jianzhu Sea	0.0008	0.0006	0.00297	0.0005
	Sparks Sea	0.0002	0.0007	0.00442	0.0010
	Panda Sea	0.0002	0.0002	0.00122	0.0006
	Wuhua Sea	0.00011	0.00000	0.00000	0.00000
Bare land	Jianzhu Sea	0.0039	0.0017	0.00765	0.0017
	Sparks Sea	0.0010	0.0041	0.01013	0.0031
	Panda Sea	0.0014	0.0007	0.00262	0.0025
	Wuhua Sea	0.00358	0.00115	0.00246	0.00095
Construction land	Jianzhu Sea	0.0015	0.0066	0.01211	0.0018
	Sparks Sea	0.0004	0.0040	0.00226	0.0010
	Panda Sea	0.0004	0.0000	0.04859	0.0051
	Wuhua Sea	0.00157	0.00279	0.00455	0.00301

## Data Availability

Not applicable.
